# Measurement of morphological changes of pear leaves in airflow based on high-speed photography

**DOI:** 10.3389/fpls.2022.900427

**Published:** 2022-11-10

**Authors:** Chao Zhang, Hongping Zhou, Linyun Xu, Yu Ru, Hao Ju, Qing Chen

**Affiliations:** College of Mechanical and Electronic Engineering, Nanjing Forestry University, Nanjing, China

**Keywords:** leaf, wind tunnel test, morphological changes, high-speed photography, motion tracking

## Abstract

The morphological changes of leaves under the airflow have a significant effect on the deposition of pesticide droplets on the leaves, but the wind-induced vibration of the leaves is complicated to measure. In this study, an aerodynamic test of the pear leaf was conducted in the wind tunnel, and binocular high-speed photography was used to record the deformation and vibration of the leaves under various airflow velocities. Experiments showed that air velocity (*v*) had a significant effect on the morphological response of the leaf. As *v* increased, the leaf was in three states, including static deformation, low-frequency vibration, and reconfiguration of airfoil steady state. The mutation from one state to another occurred at the critical velocity of *v_cr1_* and *v_cr2_*. By tracking the leaf marker point, various morphological parameters were calculated, including the bending angle of the petiole, the wind deflection angle, and the twist angle of leaves under different air velocities. When *v_cr1_* ≤*v* ≤*v_cr2_*, the parameters changed periodically. When *v*< *v_cr1_*, the petiole and the leaf bent statically, and the bending angle of the petiole and the wind deflection angle of the leaf gradually increased. When *v* >*v_cr2_*, the morphology of the leaf and the petiole was stable. Besides, this study tracked and measured the wind deflection area of leaf, which was consistent with the theoretical calculation results. The measurement of the leaf morphological parameters can reflect the morphological changes of leaves under airflow, thus providing a basis for the decision-making of air-assisted spray airflow.

## 1 Introduction

Currently, air-assisted sprayer has become an important tool for orchard pest control (Farooq and Salyani, 2008; Yang et al., 2017; Nan et al., 2022). The air-assisted spray is the process of depositing atomized droplets on the surface of leaves under high-speed airflow. On the one hand, the high-speed airflow increases the kinetic energy and the penetration distance of the droplets; on the other hand, it changes the canopy structure and increases the canopy porosity, thus widening the channel for droplets to enter the canopy and promoting the turning of leaves (Bayat and Bozdogan, 2005; Derksen et al., 2007; Llop et al., 2015). In this way, the droplets can be evenly deposited on the both side of the leaves. Under an over-low spray speed, it will be difficult for the canopy leaves to turn over, and the deposition rate of the liquid plant protection product on the back of the leaves will be reduced; under an over-high spray speed, a large number of liquid droplets will deposit to non-target areas outside the canopy, causing environmental pollution and pesticide waste (Endalew et al., 2010; Miranda-Fuentes, et al., 2018). The movement of leaves under spray has a significant effect on the deposition of droplets on leaves (Li et al., 2021). Also, the retention of pesticide droplets is closely related to the inclination of leaves. The adhesion of pesticide droplets to leaves with a small inclination can reduce the transportation and loss of pesticide droplets, and help the pesticide droplets stay on the leaves (Li et al., 2020). Under normal spray conditions, the target leaf vibrates under airflow, and the interaction between the leaves and droplets will directly affect the deposition of the droplets and the final retention of pesticide droplets on the surface of leaves. Therefore, understanding the motion of the leaves under airflow is of great significance to improving the deposition of droplets on leaves.

**Figure 1:**
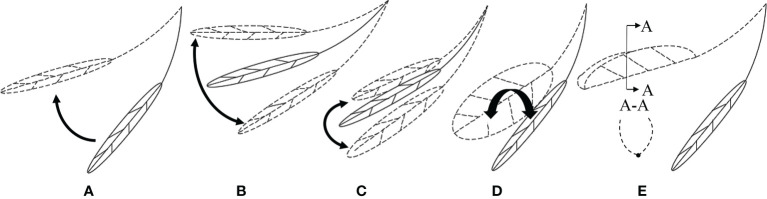
The morphological changes of the leaves in the airflow: **(A)** static lifting; **(B)** vertical swing; **(C)** horizontal swing; **(D)** torsional vibration; **(E)** wing steady.

Previous studies have shown that leaves have different mechanical behaviors under static and dynamic forces (Langre, 2019; Jiang et al., 2021). Vogel (1989) investigated individual leaves and found that when the air velocity (*v*) reaches a critical level, the leaves turn up on both sides with a U-shaped cross-section; when *v* increases to a certain level, the leaves are rolled into a cone. **Figure 1** shows the morphological changes of leaves under the airflow. Meanwhile, the movement of leaves may also be a superposition of multiple forms of motion. Miller et al. (2012) adopted particle image velocimetry technology to study the leaves of wild ginger and wild violet. The experimental results showed that when *v* was small, there was a large-scale strong vortex shedding in the leaf wake. As *v* increased, the leaves deformed, thus reducing the wind deflection area and vortex scale. Also, the increased flexibility of leaves led to increased vibration, vortex shedding, and resistance. Shao et al. (2012) conducted wind tunnel experiments on phoenix leaves. When the motion of the leaves was coupled with the wake, the leaves experienced vortex-induced vibration. After the leaves were reconfigured, a vortex could be observed behind the leaves. The lift generated by the vorticity favored the adjustment of the position and posture of the leaves, thus reducing the leaf deformation and decreasing the resistance and vibration.

Currently, contact and non-contact measurement methods are commonly used to measure vibration signals of structures. Contact measurement usually uses piezoelectric sensors to convert physical signals to voltage signals, and then converts analog signals into digital signals through data collectors. This method can obtain stable results with high measurement accuracy. However, piezoelectric sensors have obvious “additional mass effect” on light and flexible structures, and are very inconvenient to arrange the sensor on leaves. Non-contact measurement can solve the above problems. Li and Kang (2020) measured the vibration response of a single leaf to the sound. Moulia et al. (1994) investigated the static bending of corn leaves using a two-dimensional digitizing tablet. (Tadrist et al., 2014; Tadrist et al., 2015) measured the twist angle of leaves using a laser rangefinder. Meanwhile, they evaluated the inclination angle of leaves relative to the airflow direction through the motion picture of leaves taken by the monocular camera from the front. Based on this, the influence of *v* and leaf orientation on leaf flutter was analyzed. The method mentioned above simplifies the leaf motion response and cannot accurately capture the high-frequency aerodynamic response of the leaves in the airflow. With the advent of high-speed photography technology, the aerodynamic characteristics of blades under high-speed airflow can be tracked through images. Bhosale et al. (2020) used two high-speed cameras to track the 3D motion of the leaves and the tiny vibration generated by the leaves when they are hit by droplets. Then, the vibration response was decomposed into single-degree linear modes of bending and torsion.

Currently, most of the studies describe the leaf motion with visual observation or measures a single parameter on a single observation point of the leaf. These studies fail to propose a reliable measurement method for the aerodynamic response of the overall large displacement of the leaves and make an accurate numerical description of the morphological response of the leaves. In this study, the pear leaf was tested in a wind tunnel with a binocular high-speed camera. The multi-target tracking technology was used to synchronously collect the position information of multiple measuring points on the leaf. Based on this, the full-shape and multi-parameter dynamic motion response of the leaves under different air velocities was studied.

## 2 Materials and methods

### 2.1 Materials

**Figure 2:**
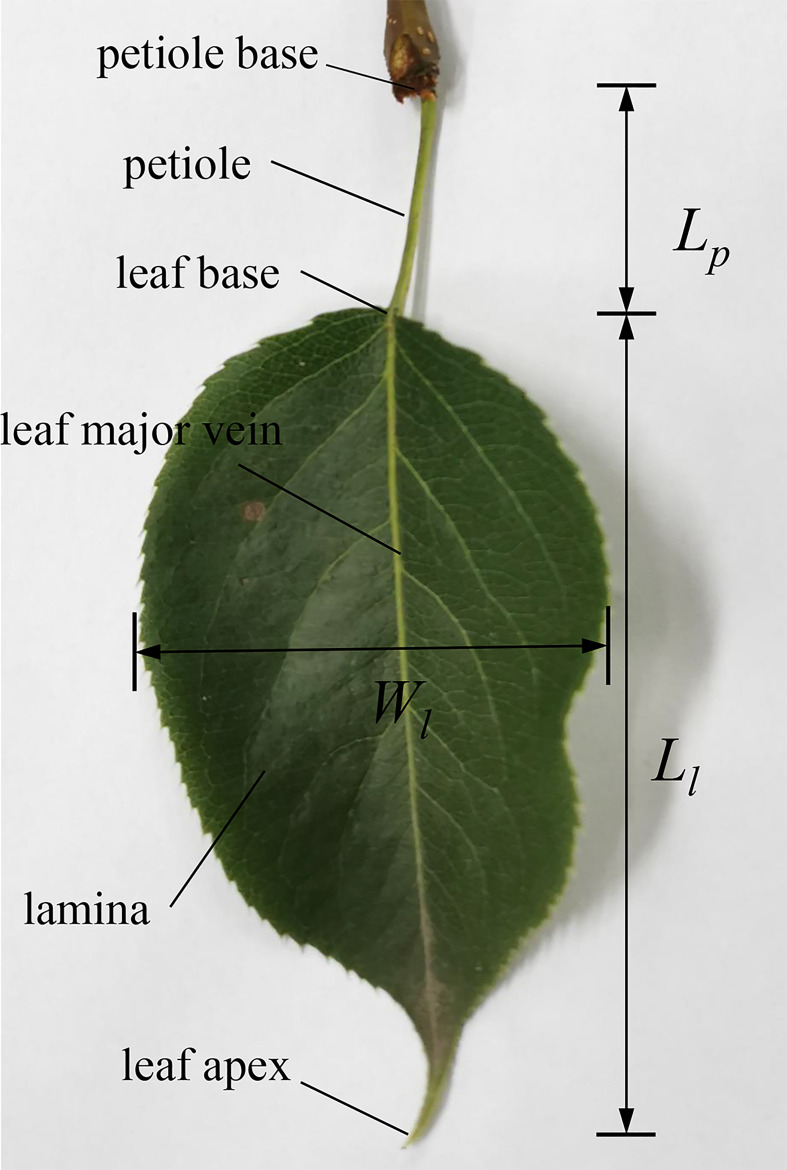
Leaf structure.

The pear is a deciduous tree and one of the main fruit crops at home and abroad. In this study, pear (*Pyrus spp*,’Sucui-I’) leaf samples were collected from Nanjing Forestry University Experimental Base in early Apr 2021. The leaf at the top of the branches was retained, and the rest of the leaves were cut off. The sample was taken from the tree and tested immediately. The collected sample leaves were heart-shaped and with a long petiole. The structure and geometric dimensions of the samples are shown in **Figure 2**, and the specific parameters are listed in **Table 1**. The length of the leaf is defined as the distance from leaf base to leaf apex, while the width of the leaf is defined as the widest distance perpendicular to the main vein of the leaf.

**Table 1:** Size of the pear leaf.

Leaf length *L*_l_/mm	Leaf width *W*_l_/mm	Leaf area *A*/mm^2^	Petiole length *P*_l_/mm	Petiole end size/mm	Petiole bottom size/mm	Average diameter *D*/mm
119	65	4687	56	2.34*1.98	2.53*1.76	2.13

The front and back of * represent the major axis and minor axis dimensions of the petiole.

### 2.2 Experiment set-up and instrument

**Figure 3:**
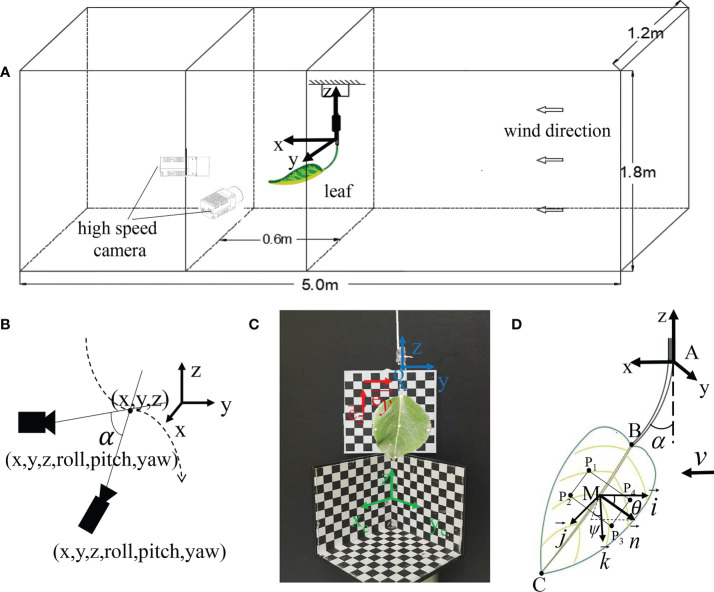
Test devices and settings : **(A)** Schematic diagram of the test sample and camera placement in a wind tunnel; **(B)** Schematic diagram of video measurement; **(C)** Coordinate calibration; **(D)** Schematic of the tracking point and different angles.

This experiment was conducted in the wind tunnel (L = 8 m, W = 1.2 m, H = 1.8 m) at Nanjing Forestry University. The air velocity was uniform and continuously adjustable in the range of 0.2-10 m/s, and the turbulence intensity was less than 0.5%. As shown in **Figure 3A**, the twig connecting the leaf was fixed with tape on a metal rod with a diameter of 0.5 mm, and it was placed vertically in the middle of the test section. When there was no wind, the leaf drooped naturally, and the surface of the leaf should be as vertical as possible to the wall of the wind tunnel. During the test, the metal rod did not vibrate or deform under the wind tunnel airflow. Meanwhile, *v* was increased from 0 to 8 m/s by a step of 0.5 m/s. The experiment was finished within 30 min so that the leaf showed no dehydration or wilting, and the flexibility of the petiole did not change over time.

Two high-speed cameras (M310 and VEO410) from Vision Research in the United States were used to shoot and track the movement of the leaf. The minimum exposure time of M310 is 1 μs, and the maximum resolution is 1600×1200; the minimum exposure time of VEO410 is 1 μs, and the maximum resolution is 1280×800. The Phantom supporting software Pcc was used to obtain the images synchronously with Nikon’s AF Zoom-NIKKOR 24-85 mm f/2.8-4D zoom lens. The high-speed camera was facing the leaf so that the leaf was in the center of the picture, and the focus was adjusted to make the picture clearest. The image resolution of the camera was set to 1280×720, and the shooting rate was 500 fps. A calibration plate was used to calibrate the 3D coordinates before the experiment was conducted. Since the high-speed camera was placed in the wind tunnel, it could affect the airflow stability. To address this issue, the high-speed camera was located 1 m away from the test device. The wind tunnel air velocity was adjusted by the fan speed of the wind tunnel. Besides, a hot wire anemometer was fixed at the installation position of the fan to measure the disturbance of the test device on the air velocity. Under all air velocities, the relative errors of the measuring points were less than 1%, indicating that the disturbance caused by the test device was negligible.

### 2.3 Camera calibration and marker point tracking

The coordinates of the marker points on the leaf were tracked by TEMA Motion (Image Systems AB, Sweden). TEMA Motion applies the concept of intersection to the analysis in 3D space through two high-speed cameras (Xuan et al., 2020). The target observations (tracked 2D pixel coordinates) and the camera pose were used to calculate the 3D position of the target. Meanwhile, the camera pose was calibrated, and several common points in the camera view were taken as references (3D calibration objects in **Figure 3C**). Then, the scale was added, and there was no need to add coordinate measuring equipment in the measurement process. **Figure 3B** shows the schematic diagram of the video measurement. Before the test, the 3D checkerboard calibration object was placed at the measurement position of the leaf, and its placement position should make sure that the calibration grid was located in the two high-speed camera windows, which was used as a coordinate reference for the software to track the marker point. Herein, the target position to be measured was marked with a marker on the leaf. The marker point must always be located in the camera window during the measurement. TEMA Motion can automatically track the 3D coordinates of the target position in the calibration reference system.

To make the reference system consistent with the defined one, a 2D checkerboard square calibration paper was added, and the vertical direction and horizontal direction of the paper were respectively the z-axis and the y-axis of the defined coordinate system. The direction vectors $\vec{e_{z}}$ and $\vec{e_{y}}$ of the z direction and the y direction were respectively determined by two points in the vertical and horizontal directions on the chessboard. The x-direction is the wind tunnel airflow direction, and the direction vector is ${\vec{e}}_{x}=\vec{e_{y}}\times \vec{e_{z}}$ By solving the rotation attitude angle of the vector in each direction of the defined coordinate system and the reference coordinate system, the rotation angle in each direction and the rotation matrix were obtained. Then, each tracking point was converted from the calibrated coordinate system to the set coordinate system through the coordinate conversion matrix:


(1) $$\left(\begin{array}{ccc} x_{id} & y_{id} & z_{id} \end{array}\right)=\left(\begin{array}{ccc} x_{ir} & y_{ir} & z_{ir} \end{array}\right)M_{rotation}+M_{offset}$$

where (*x_id_ y_id_ z_id_*) is the coordinate of each point in the custom reference system; (*x_id_ y_id_ z_id_*) is the coordinate of each point in the reference coordinate system; *M_rotation_* is the coordinate rotation matrix, and *M_offset_* is the coordinate offset matrix.

### 2.4 Determination of the spatial location of the leaf

The position and attitude of the leaf in the airflow are important parameters that affect the capture of droplets. The tracking points were marked on the leaf. Specifically, A is the connection between the petiole and the branch; B is the position of leaf base; C is the position of leaf apex; M is the center of the leaf, and *P*_1_-*P*_4_ are the four points on the plane of the middle position of the leaf. The posture change of the leaf can be determined by spatial vector calculation (**Figure 3D**).

The bending angle α of the petiole is defined as the angle between the petiole and the vertical direction. Assuming that under low-speed airflow, the leaf does not curl up or bend, and the leaf plane is regarded as a rigid body plane. The coordinate system Mx'y'z' is the leaf follower coordinate system, where x' is opposite to the airflow direction; z' is the vertical direction, and the downward direction is the positive direction. $\vec{n}$ is the normal vector of the leaf plane obtained by P1-P4, $\vec{n}=\vec{P_{1}P_{3}}\times \vec{P_{2}P_{4}}$ . The wind deflection angle of the leaf (θ) is defined as the angle between the normal vector of the leaf and the wind direction. The twisting azimuth angle *ψ* of the leaf is the angle between the projection of the normal vector $\vec{n}$ on the vertical plane My'z' and the vertical axis Mz', and it is positive in the clockwise direction. When the leaf is bent and curled, the leaf cannot be treated as a plane. In this case, the wind deflection angle of the leaf can be approximated as the angle between the vector $\vec{BC}$ and the airflow direction.

### 2.5 Wind deflection area of leaf

**Figure 4:**
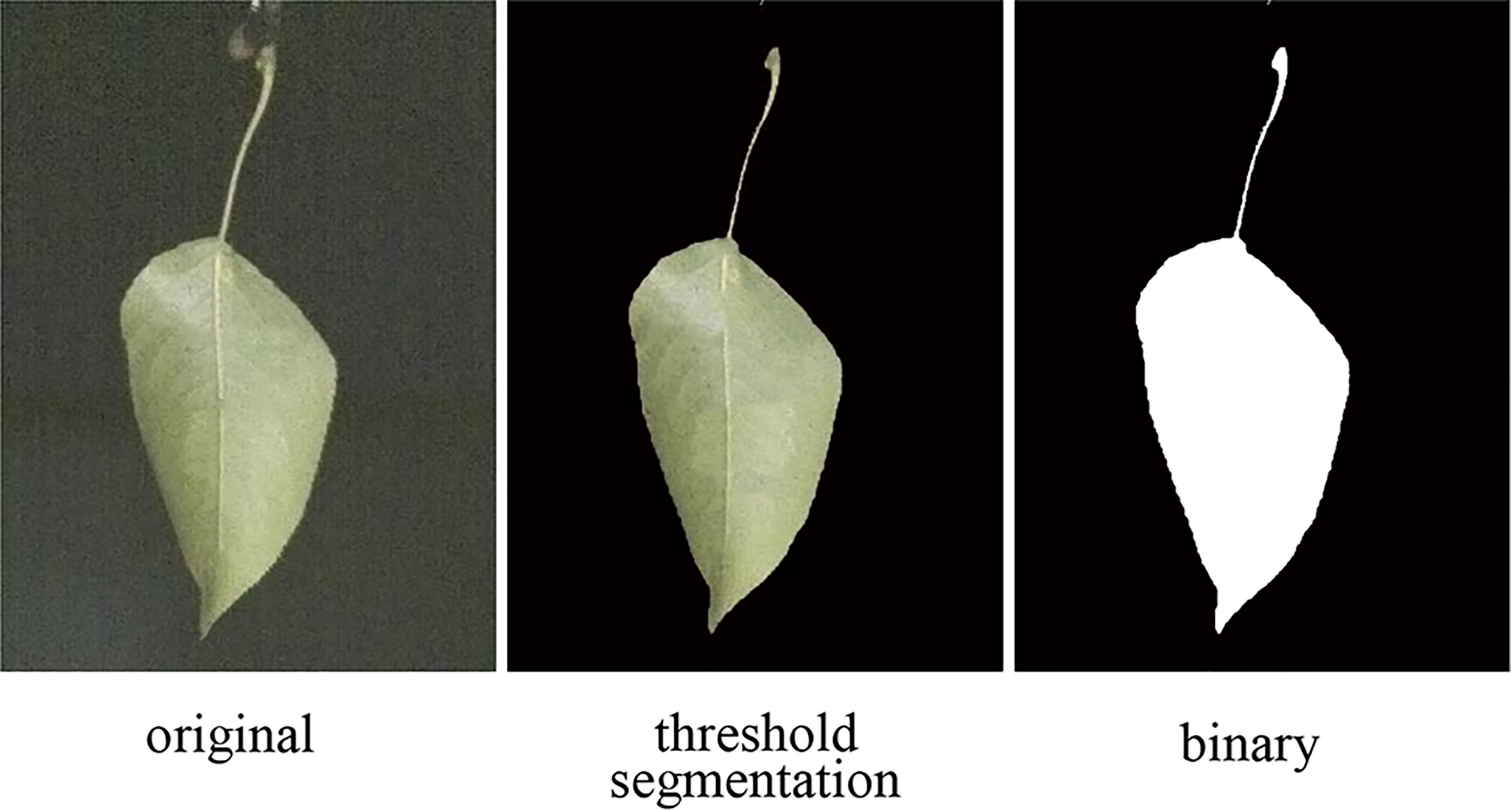
Image processing to obtain the wind deflection area of the leaf.

The wind deflection area is the projected area of the leaf in the direction of the airflow. The image processing method (Prasad and Singh, 2017) can be exploited to capture the changes of the area of the leaf moving in the airflow. **Figure 4** shows the image processing flow. First, the RGB format image taken by the high-speed camera was converted to HSV (hue, saturation, value) format, and the HSV color model combined with the OpenCV library was used to segment the image. Then, the checkerboard calibration plate image was segmented to obtain the area represented by the unit pixel. Next, the green threshold interval of the pear leaf in the HSV color model was used to extract the leaf from the background. After that, the image was binarized, and the wind deflection area of the leaf was calculated by the pixel counting method, and the black and white square calibration plate with known size (1cm × 1cm) was used for scaling.

The wind deflection area of the leaf can be calculated by Eq. (2):


(2) $$A_{f}=\frac{S_{label}}{N_{label}}N_{plant}$$

Where *A_f_* is the actual projected area of the leaf (cm^2^); *S_label_* is the actual area of the calibration plate (1 cm^2^); *N_label_* and *N_plant_* are the number of pixels in the calibration plate and the projected area of the leaf in the binary image, respectively.

## 3 Results

### 3.1 Leaf vibration state under wind

**Figure 5:**
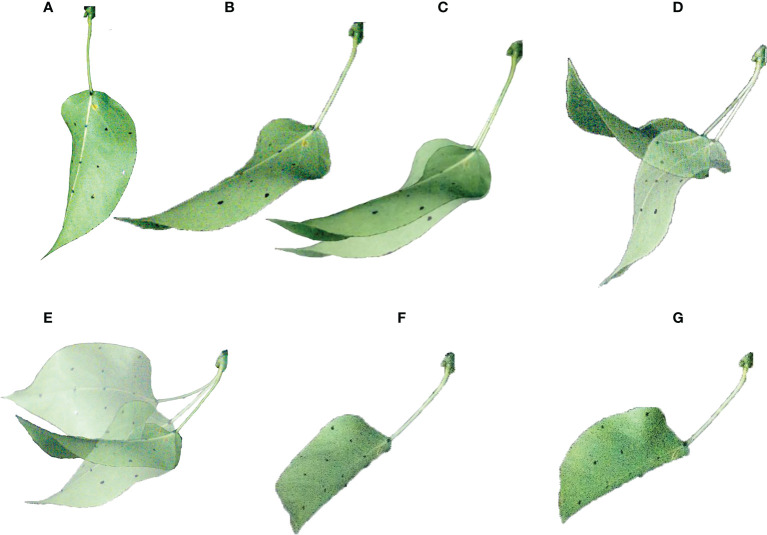
Deformation and vibration of the leaf under different air velocities. **(A)** *v*=0m/s; **(B)** *v* =2.0m/s; **(C)** *v* =2.5m/s; **(D)** *v* =3.0m/s; **(E)** *v* =3.5m/s; **(F)** *v* =4.0m/s; **(G)** *v* =8.0m/s.

The leaf of the pear tree was hung in the middle of the test section of the wind tunnel, and the deformation of the leaf under the increase of *v* was observed. **Figure 5** shows the vibration and deformation of the pear leaf under different air velocities.

The motion response of the leaf under airflow is the same as that listed in **Figure 1**. As shown in **Figure 5A**, when *v*< 2.5 m/s, as *v* increased, the leaf slowly rose, and the inclination angle of the leaf increased. Also, the leaf kept still under a stable air velocity. As shown in **Figure 5C**, when *v* = 2.5 m/s, the leaf started to vibrate at a low frequency, and the petiole was bent and twisted. The velocity that causes the change of leaf motion shape was defined as critical air velocity, and the air velocity at this time is defined as the first critical velocity *v_cr_*_1_. As the vibration amplitude gradually increased, the form of the vibration was up-down and left-right swing, and the petiole was bent and twisted. As shown in **Figure 5E**, when *v* = 3.5 m/s, the leaf drove the petiole to have a large torsion and up-down vibration, and the leaf flipped around the connection point of the petiole and the leaf. As shown in **Figure 5F**, when *v* = 4 m/s, the leaf stopped swinging, but the petiole continued to bend. The left and right parts of the leaf bent toward the main stem of the leaf to form a “U” shape, and the leaf was twisted. At this time, the leaf changed from a large vibration to a stable state, and the air velocity was defined as the second critical velocity *v_cr_*_2_. As *v* increased, the bending of the petiole and the leaf increased, and high-frequency vibration occurred at the leaf apex.

**Figure 6:**
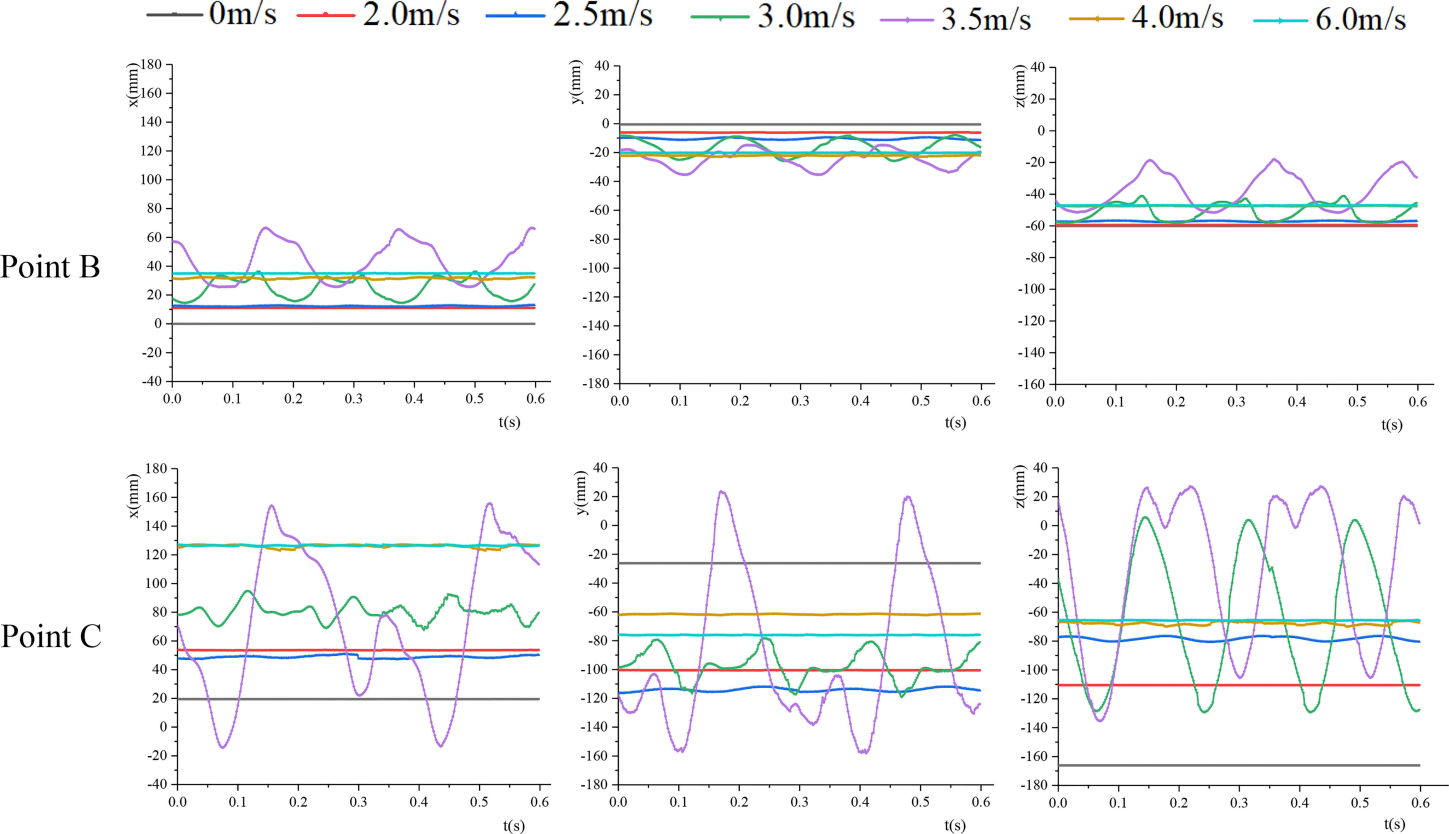
The coordinate changes of points B and C in each direction.

The 3D coordinate point of the marker point on the leaf was tracked by TEMA Motion software. A total of 300 frames of photos were tracked, and the 3D coordinates of the marker point were recorded. The joint A of the petiole and the branch was taken as the origin of the coordinates, and no perceptible movement occurred during the test. **Figure 6** shows the coordinate changes of leaf base point B and leaf apex C in each direction under different air velocities. According to the analysis of the coordinates of each point, within the range of 0-2 m/s, the leaf coordinates were stable. Also, no vibration occurred, and the leaf was in static deformation. Due to the non-uniform change of the cross-section of the petiole and the initial position relationship between the leaf and the petiole, the static bending directions of the petiole and the leaf were mainly along the airflow direction, and there was also a small displacement in the y-direction. The petiole and the leaf moved in the space to find a static equilibrium position. As *v* increased, the petiole tended to bend in the airflow direction. Within the range of the critical velocities, both the petiole and the leaf moved periodically.

The vibration frequencies and amplitudes of points B and C calculated by FFT (Fast Fourier Transform) are listed in **Table 2**. When the velocity was slow, the vibration frequencies of point B and point C were basically the same, and the vibration frequencies of the two points in the x, y, and z directions were basically the same. When *v*=3.5m/s, the vibration frequencies of both point B and point C decreased, and there was a great difference in the vibration frequencies of point C in each direction. The vibration frequency in the z direction was significantly higher than that in the x and y directions, and it was consistent with the vibration frequency in the z direction of point B. The vibration frequency of leaf base point B was greater than that of leaf apex point C. As the vibration amplitude increased, the vibration frequency decreased. When *v* >*v_cr_*_2_, the coordinates of the leaf apex and leaf base were basically unchanged under different air velocities, and the leaf tended to be stable. As *v* increased, the coordinate changes in each direction were small.

**Table 2:** Vibration frequency and amplitude of points B and C at *v*=3.0m/s and *v*=3.5m/s.

Velocity (m/s)	Direction	Point B		Point C	
		Frequency (Hz)	Amplitude (mm)	Frequency (Hz)	amplitude (mm)
3	x	5.6	8.29	5.8	3.28
	y	5.6	7.65	5.8	8.78
	z	5.6	4.92	5.8	46
3.5	x	4.6	17.72	2.8	36.16
	y	4.6	8.17	3.4	60.44
	z	4.6	13.39	4.8	54.21

### 3.2 Morphological changes of the leaf

#### 3.2.1 Wind deflection angle and twist angle of the leaf

**Figure 7:**
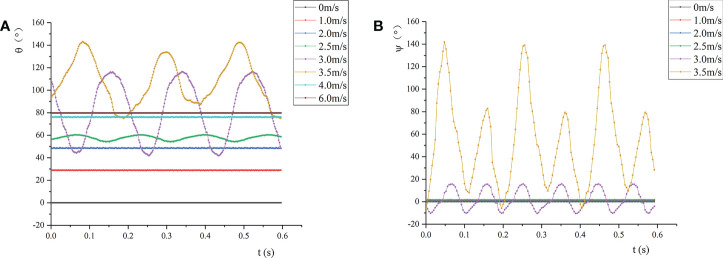
Wind deflection angle and twist angle of the leaf at different air velocities: **(A)** Wind deflection angle; **(B)** twist angle.

**Figure 7** shows the change of the wind deflection angle and twist angle of the leaf under airflow. By definition, the wind deflection angle can be approximately regarded as the bending angle of the leaf in the direction of the airflow (**Figure 7A**). When *v* reached the first critical velocity, the wind deflection angle of the leaf changed periodically. Meanwhile, the vibration amplitude increased with the increase of *v*, and the frequency gradually decreased. When *v* approached the second critical velocity, although the wind deflection angle changed periodically, its average and amplitude both reached the maximum. When *v* >*v_cr_*_2_, the wind deflection angle decreased and was relatively stable. Afterwards, as the air velocity increased, the wind deflection angle increased slightly and approached 90°, that is, the leaf tended to bend in the direction of the airflow. This is because the leaf curled up into a cone, which reduced the airflow resistance through the reconstruction of its own shape to alleviate the damage of the airflow to the plant organs. The reconstruction of the leaf reduced the drag force of the airflow on the leaf, and the bending of the leaf did not vary significantly.

**Figure 7B** shows the change of the twist angle of the leaf. Under the test conditions, when *v*< *v_cr1_*, the twist angle was almost 0, and the leaf did not twist. When *v*= 2.5 m/s, the wind deflection angle of the leaf changed periodically. At this time, the twist angle of the petiole was almost 0, indicating that the torsional and bending vibrations of the leaf did not occur simultaneously. As *v* gradually increased, the leaf presented torsional vibration. When *v_cr_*_1_≤ *v* ≤*v_cr_*_2_, the torsion of the leaf intensified with the increase of *v*, which changed from small amplitude of high frequency to large amplitude of low frequency. Also, the torsional vibration frequency of the leaf was greater than the vertical vibration frequency of the leaf. When *v*= 3.0 m/s, the amplitudes of the wind deflection angle and twist angle of the leaf were respectively 59.7° and 26.1°, and the vibration was dominated by vertical vibration. When *v*= 3.5 m/s, the amplitudes of the wind deflection angle and twist angle of the leaf were 68.3°and 146.1°, respectively; at this time, the vibration was dominated by torsional vibration. When *v* >*v_cr_*_2_, the leaf was bent toward the veins, and the leaf cannot be treated as a flat surface. It was found through experiments that in the range of the test air velocity, when *v* >*v_cr_*_2_, the leaf hardly twisted, so it can be considered that the twist angle remained unchanged.

#### 3.2.2 Wind deflection area of leaf

**Figure 8:**
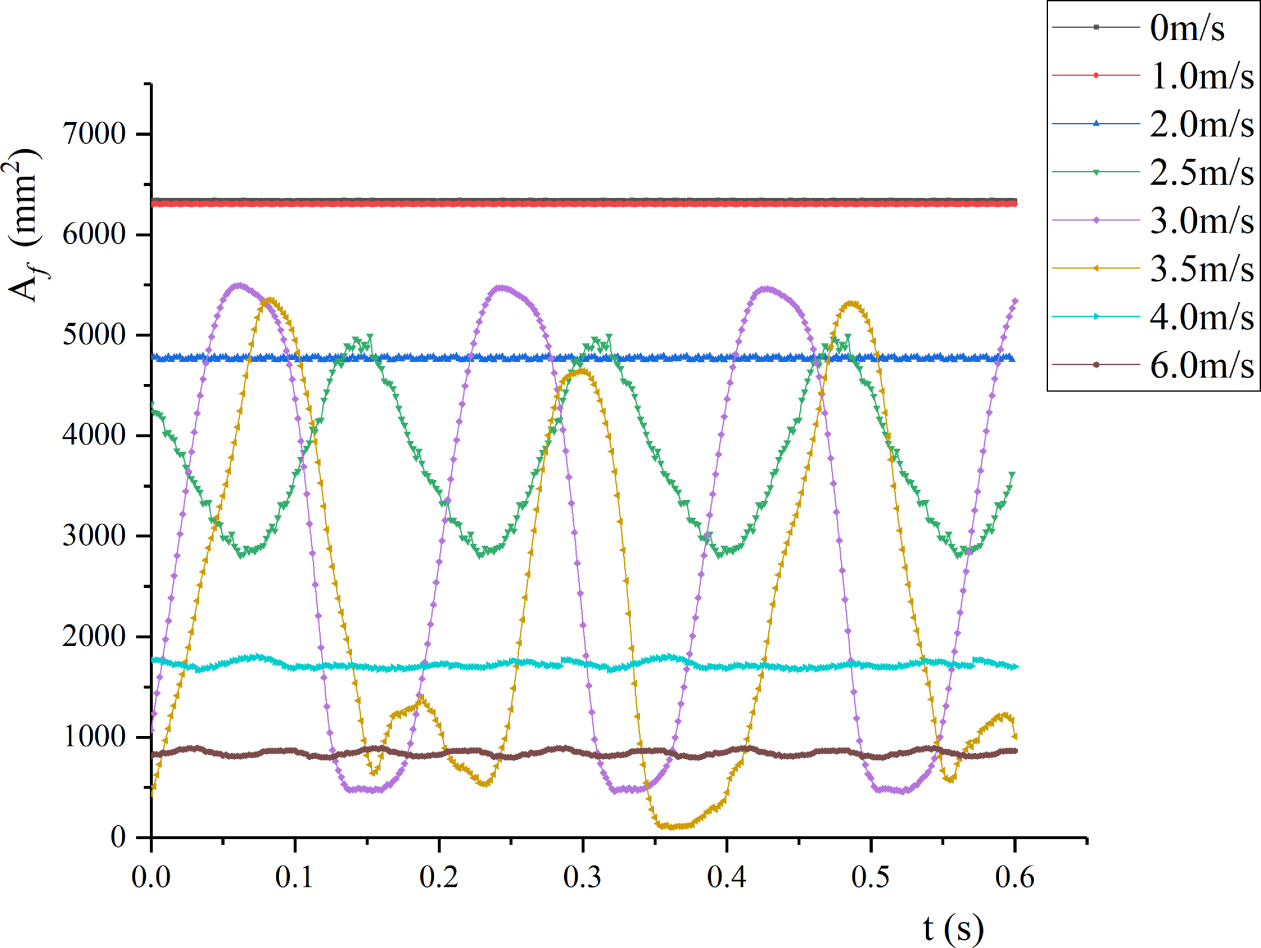
Wind deflection areas of pear leaf under different air velocities.

By segmenting the high-speed camera facing the leaf in the wind tunnel, the wind deflection area under time-averaged air velocity was calculated. **Figure 8** shows the wind deflection area under different air velocities (0-6 m/s).

As *v* increased, the average wind deflection area decreased. At low air velocities, the leaf was in a stable state, and the wind deflection area remained basically constant. When *v*< 1 m/s, the leaf area was basically unchanged, and the pressure generated by the airflow failed to overcome the inertial force of the leaf itself. When *v* > 1 m/s, as *v* increased, the wind deflection area of the leaf gradually decreased. When *v*< *v_cr_*_1_, the change of the wind deflection area of the leaf was mainly caused by static bending. When *v_cr_*_1_≤ *v* ≤*v_cr_*_2_, the leaf presented low-frequency vibration, and the leaf area changed periodically; as *v* increased, the vibration amplitude and period gradually increased. When *v* = 3.5 m/s, the leaf tended to rotate around the petiole. Under *v* = 3 and 3.5 m/s, the leaf area changed periodically with a period of 0.19 and 0.42 s, respectively. When *v* = 4 m/s, the wind deflection area decreased sharply to 27% of the initial leaf area, and the leaf area fluctuated slightly. As *v* increased, the leaf gradually lifted, and the leaf area decreased.

### 3.3 Petiole bending

**Figure 9:**
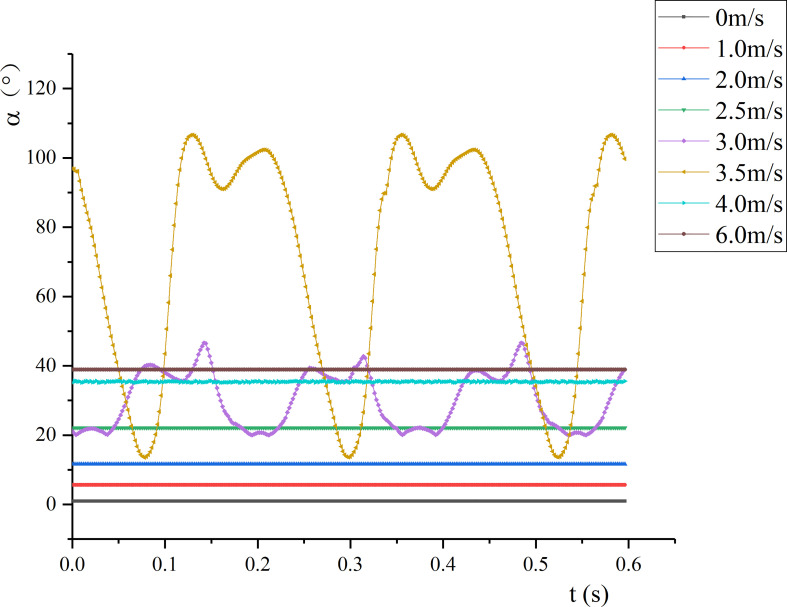
Petiole bending angle.

Under time-averaged force, petiole presented bending deformation. The vertical angle changes under each air velocity were calculated through the coordinates (see **Figure 9**). As *v* increased, the angle between the petiole and the vertical direction gradually increased. When *v_cr_*_1_≤ *v* ≤*v_cr_*_2_, the bending of the petiole changed periodically; as *v* increased, the variation amplitude increased but the variation frequency decreased. By comparing the bending angle of the petiole (α) with the wind deflection angle of the leaf (θ), it can be seen that θ changes more than α in the case of static bending because the bending strength of the leaf was lower than that of the petiole. When *v* = *v_cr_*_1_, the petiole’s bending was stable, and the leaf swung before the petiole around the base position. When *v_cr_*_1_≤ *v* ≤*v_cr_*_2_, the change amplitude of the petiole’s bending gradually increased, and the change frequency decreased. The changes in the bending angle of the petiole and the wind deflection angle of the leaf were asynchronous, and the peak value of the wind deflection angle of the leaf was always greater than that of the bending angle of the petiole.

## 4 Discussion

The leaf morphology was measured by high-speed photography, and there was a correlation between the leaf morphology parameters. The validity of the measurement method and the authenticity of the test results were verified by discussing the relationship between the morphological parameters.

### 4.1 Leaf critical velocity and morphology changes

To verify the universality of the test phenomenon, a wind tunnel test was performed on 30 leaves in the same period. The 30 experimental leaves were selected randomly from the pear trees in the experimental park. The leaves were tested in the wind tunnel according to the method described in this paper. The test results showed that the first critical air velocity of pear trees was 2.5-3.5m/s, and the second critical air velocity was 3.0-5.0m/s. (Shao et al., 2012; Shao et al., 2017) studied sycamore leaves and tulip tree leaves. The values of *v_cr_*_1_ were 2.0 and 3.7 m/s, and the values of *v_cr2_* were 3.4 and 5.6 m/s, which were different from the critical air velocities of the pear leaf investigated in this study. The critical air velocities of leaves of different tree species are quite different, which is related to the physiological characteristics of the leave. The critical velocity of different leaves of the same trees species will also be different, but the critical air velocity of the leaves of the same species changes within a small range.

Meanwhile, the air velocity range of some leaves from static deformation to airfoil steady state was only 0.5 m/s, and the state change was extremely rapid. The factors that affect the critical air velocity of the leaf include the shape and size of the leaf and the petiole and the material properties, which are not discussed in this paper. Among the 30 leaves under test, 30% of the leaves experienced all the morphological changes shown in **Figure 5**, the other 70% presented large torsional vibration instead of significant vertical vibration (**Figure 5D**). This may be attributed to the over-large change interval of the air velocity.

### 4.2 Wind deflection area and deflection angle

**Figure 10:**
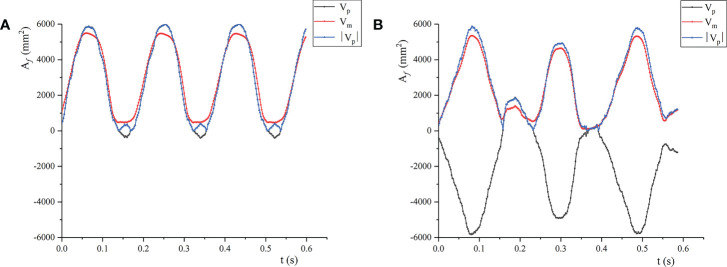
Predicted and measured values of leaf area change: **(A)** *v* = 3.0m/s; **(B)** *v* = 3.5m/s; Vp is the predicted value calculated by the wind deflection angle; Vm is the actual measured value; |Vp| is the absolute value of the predicted value.

The wind deflection area under airflow satisfies *A*_f_ = *A*_0_ cos *θ* at *v*< *v_cr_*_1_. For the case where the leaf movement varied greatly (*v* = 3.0 and 3.5 m/s), the changes of the calculated value and the measured value are shown in **Figure 10**. For the calculated value, the leaf area under the two air velocities was negative, indicating that the windward side of the leaf changed from the front to the back, and the front and the back became windward alternately. When *v* = 3.0 m/s, the front as the windward side accounted for 82% of the cycle; When *v* = 3.5 m/s, the front as the windward side accounted for 20% of the cycle. The results showed that calculating the wind deflection area of the leaf by image processing has limitations and cannot distinguish the windward surface of the leaf. By contrast, the wind deflection area of the leaf solved by the wind deflection angle can reflect the windward surface of the leaf well. The correspondence between the calculated and the measured wind deflection areas demonstrates the reliability of the binocular video measurement method in measuring the leaf motion shape.

### 4.3 Static bending of petiole

**Figure 11:**
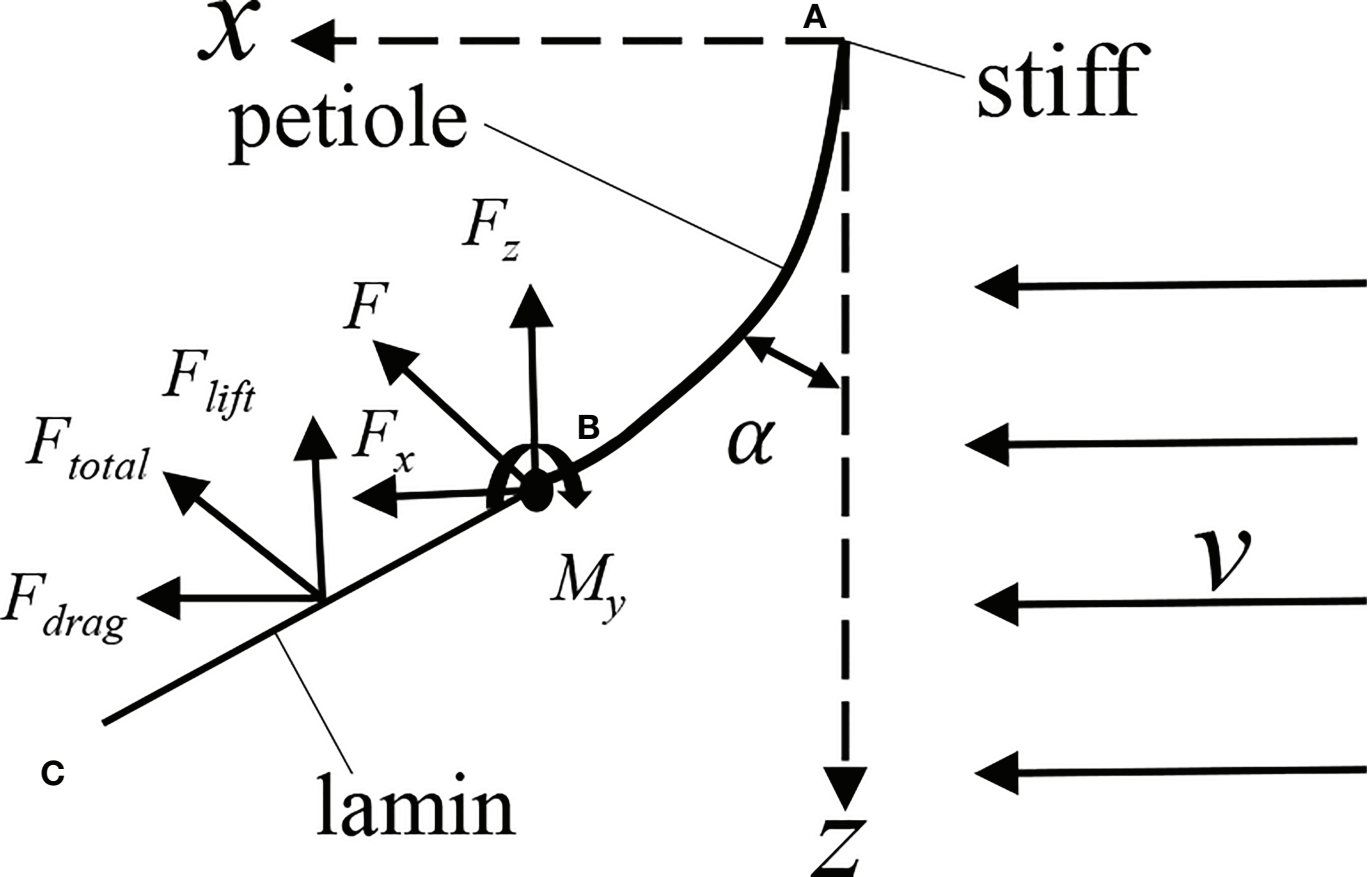
Analysis of leaf force. **(A)**, position of the petiole base; **(B)**, position of leaf base; **(C)**, the position of leaf apex.

As shown in **Figure 11**, under time-averaged force, the petiole presented bending deformation, and the aerodynamic force received by the leaf can be divided into flow force *F_drag_*, vertical upward lift force *F_lift_*, and lateral force *F_y_*. Since the lateral force is small, it can be ignored. Ignoring the mass force of the leaf, the forces acting on the free end of the petiole can be expressed as *F_x_* = *F_drag_* and *F_z_* = *F_lift_*, and the lateral force can also be ignored. The leaf was also subject to the moments in three directions. Due to the large moment *M_y_* of *F_drag_* in the horizontal direction on the y-axis, the petiole mainly bent in the xoz plane, and the moments in other directions were small and can be ignored. The petiole can be simplified as a cantilever beam, where one end is fixed to the wall, and the other end is free and subject to concentrated forces and moments.

During static deformation, the bending angle α of the petiole at a certain point on the xz plane can be described by the following equation (Luan and Yu, 1991; Shao and Zhu, 2017):


(3) $$\begin{array}{c} \frac{\text{d}\alpha }{\text{d}s}=\frac{M_{y}+F_{x}(1-z_{m}-z)+F_{z}(x_{m}-x)}{EI}\\ \frac{\text{d}z}{\text{d}s}=\cos\alpha ,\frac{\text{d}x}{\text{d}s}=\sin\alpha \end{array}\}$$

where *α* is the bending angle of the petiole; *s* is the arc length (0≤*s*≤*L*_*p*_) ; *x* and *z* are the dimensionless rectangular coordinates of the point; *x_m_*, *z_m_* are the coordinate values of the end of the petiole; *F_x_* and *F_z_* are the component forces of the aerodynamic force acting on the petiole in *x* and *z* directions, respectively; *E* is the elastic modulus of the petiole, MPa. Following the measurement method in (Shah, Reynolds and Ramage, 2017), the measured elastic modulus of the leaf is *E*=125 MPa; $I=\frac{\pi D^{4}}{64}$ is the inertia moment of the cross-section of the petiole, and the cross-section is elliptical. To simplify the calculation, the diameter of the petiole was replaced by the average diameter, *D*=2.13mm.

The resistance of the leaf can be expressed as $F_{drag}=C_{d}\frac{\rho v^{2}}{2}A_{f}$ , where *C_d_* is the resistance coefficient of the leaf. According to the literature (Stanford and Tanner, 1985), *C_d_* ≈1.2; *ρ* is the air density, and *ρ*=1.293kg/m^3^ ; *v* is the air velocity in the wind tunnel, and *A*_*f*_ is the windward area of the leaf.

The lift acting on the leaf can be expressed as $F_{lift}=C_{l}\frac{\rho v_{a}^{2}}{2}A_{f}$ , where *C_l_* is the lift coefficient, and $C_{l}=\sin2(\frac{\pi }{2}-\theta )$ (Jiang et al., 2011).

Assuming that the acting point of the aerodynamic force on the leaf is close to the position of the middle length of the leaf, we have


(4) $$M_{y}\approx \frac{L_{l}}{2}(F_{drag}\cos\theta +F_{lift}\sin\theta )$$

where *L_l_* is the leaf length, and *θ* is the angle between the leaf and the yoz plane, i.e., the wind deflection angle. Taking the derivative of s at both ends of the equation, according to the relationship between the derivative of *x* and *z* with respect to *s*, the following equation can be obtained:


(5) $$\frac{{\text{d}}^{2}\alpha }{\text{d}s^{2}}\text{=-}\left(a\cos\alpha +b\sin\alpha \right)$$

where $a=\frac{F_{x}l^{2}}{EI},b=\frac{F_{z}l^{2}}{EI}$ .

**Figure 12:**
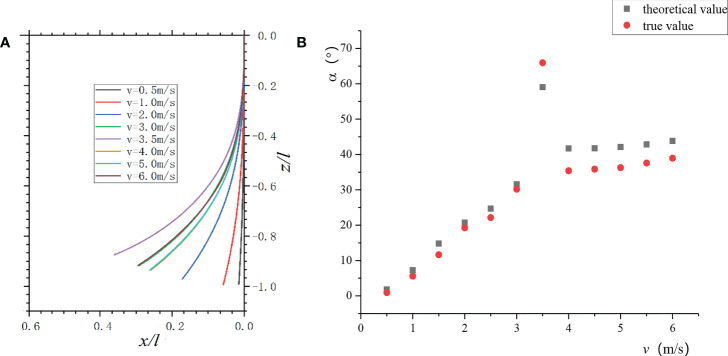
Petiole bending vs. *v*: **(A)** Change of a petiole bending with air velocities; **(B)** The theoretical and measured values of petiole’s bending under each air velocity.

The numerical solution of the above ordinary differential Eq. (5) was solved, and the petiole curve under each air velocity was obtained according to the petiole geometric relationship (**Figure 12A**). When 0< *v*< *v_cr1_*, the petiole’s bending changed rapidly; when *v* >*v_cr2_*, the petiole’s bending decreased, and the bending changed slowly with the increase of *v*, which was consistent with the experimental observation.

The theoretically calculated value was compared with the actual measured value of the petiole’s end bending. As shown in **Figure 12B**, at low air velocities, the theoretical value of the petiole’s static bending was more consistent with the actual value. When *v_cr_*_1_≤ *v* ≤*v_cr_*_2_, although the bending angle changed periodically, the average in the period had a good correlation with the theoretical value. When *v* = 3.5 m/s, the theoretical value deviated significantly from the actual value because the torsional vibration of the leaf increased the complexity of the leaf-stalk system. When *v* >*v_cr_*_2_, the leaf curled into a wing with curvature, and the airflow passed through the wing to form a circulation. Thus, the measured values were less than the theoretical values.

## 5 Conclusions

In this study, an aerodynamic test of apear leaf was conducted in a wind tunnel, and binocular high-speed photography was used to record the deformation and vibration of the leaf. The test showed that when the front of the leaf was windward, the leaf experienced static deformation under low-speed airflow, and the leaf rose slowly; when the air velocity reached the first critical air velocity *v_cr1_*, the leaf presented up-down flapping vibration at a low frequency and a large amplitude. As *v* increased, the leaf presented torsional vibration around the junction of the leaf and the petiole. When the air velocity reached the second critical velocity, the leaf was curled up, and it stopped vibration to be in a stable state.

As the air velocity increased, the wind deflection area of the leaf gradually decreased. When *v_cr_*_1_≤ *v* ≤*v_cr2_*, the wind deflection area of the leaf changed periodically. During the vibration period, the front and back of the leaf became the windward side alternately, which increased the probability of the deposition of droplets on the front and back sides of the leaf. The change of the wind deflection area of the leaf tended to be slow and finally stabilized at the minimum when *v* >*v_cr_*_2_.

By tracking the characteristic points on the pear leaf, the vector analysis method was adopted to calculate the bending angle of the petiole and the wind deflection angle, and the twist angle of the leaf under airflow. When *v*< *v_cr_*_1_, the petiole experienced static bending, and the leaf was lifted. When *v_cr_*_1_≤ *v* ≤*v_cr_*_2_, the bending angle of the petiole and the wind deflection angle of the leaf changed periodically, and the change amplitude and average increased with the increase of *v*. Meanwhile, the leaf exhibited periodic torsional vibration. During the vibration period, the wind deflection angle was greater than 90°, and the front and back of the leaf became the windward side alternately. When *v* >*v_cr_*_2_, the bending angle of the petiole and the wind deflection angle of the leaf were relatively stable, and they increased slightly with the increase of *v*. The leaf eventually tended to be in the direction of airflow. Regarding the petiole as the cantilever beam model, this study converted the aerodynamic forces into concentrated loads and bending moments, and the bending deformation of the petiole under airflow was derived theoretically. When *v_<_ v_cr_*_2_, the petiole’s bending changed fast. When *v* >*v_cr_*_2_, the petiole’s bending decreased, and the change tended to be slow.

In this paper, we proposed a method for measuring the motion response of leaves under the action of airflow, and given the test results. In addition to airflow, leaf parameters such as the leaf elastic modulus, shape and size, maturity, etc. have important impact on leaf movement response. Subsequent research can be carried out on the influence of the physiological parameters of pear leaves on their movement response.

## Data availability statement

The raw data supporting the conclusions of this article will be made available by the authors, without undue reservation.

## Author contributions

CZ, HZ, and LX conceived the study. CZ, HJ, and YR performed the experiments. CZ analyzed the data. CZ prepared the figures. CZ, HZ, YR, and QC drafted the manuscript. All authors discussed the results,edited the manuscript, and approved the final manuscript.

## Funding

This work was supported by the National key Research and Development Program of China (2018YFD0600202-04) and National Natural Secience Foundation of China (51906111).

## Acknowledgments

The authors acknowledge the support of Nantong Guangyi Mechanical & Electronical Co., Ltd during the experimental set-up.

## Conflict of interest

The authors declare that the research was conducted in the absence of any commercial or financial relationships that could be construed as a potential conflict of interest.

## Publisher’s note

All claims expressed in this article are solely those of the authors and do not necessarily represent those of their affiliated organizations, or those of the publisher, the editors and the reviewers. Any product that may be evaluated in this article, or claim that may be made by its manufacturer, is not guaranteed or endorsed by the publisher.
