# Supplementary material for: Measurement of morphological changes of pear leaves in airflow based on high-speed photography
Source: Front Plant Sci. 2022 Nov 10;13:900427. doi: 10.3389/fpls.2022.900427 (PMC9685665; doi:10.3389/fpls.2022.900427)
Supplement: Supplementary file 1 [file DataSheet_1.docx]

Supplementary Material

# Supplementary Data

# Supplementary Figures and Tables

**Table 1. Air velocities at the measuring points with/without test device under different fan speeds**

| Fan speed (r/min) | Air velocity without test device (m/s) | Air velocity with test device (m/s) | The relative errors (%) |
| --- | --- | --- | --- |
| 8 | 0.99 | 0.98 | 1.0 |
| 14 | 2.03 | 2.01 | 1.0 |
| 20 | 2.98 | 2.97 | 0.3 |
| 26 | 4.01 | 3.99 | 0.5 |
| 32 | 5.0 | 4.98 | 0.4 |
| 38 | 5.97 | 5.96 | 0.2 |
| 44 | 6.96 | 6.94 | 0.3 |
| 50 | 7.97 | 7.94 | 0.4 |

Table 2. Statistics of 30 leaf samples morphological changes in airflow

| Leaf number | $v_{cr1}$(m/s) | $v_{cr2}$(m/s) | vertical vibration | torsion vibration |
| --- | --- | --- | --- | --- |
| 1 | 2.5 | 3.5 | Yes | Yes |
| 2 | 2.5 | 3.0 | No | Yes |
| 3 | 2.5 | 3.0 | No | Yes |
| 4 | 3.5 | 4.5 | No | Yes |
| 5 | 3.5 | 4.0 | No | Yes |
| 6 | 3.5 | 5.0 | No | Yes |
| 7 | 2.5 | 3.5 | Yes | Yes |
| 8 | 3.0 | 4.5 | Yes | Yes |
| 9 | 3.0 | 3.5 | No | Yes |
| 10 | 3.5 | 4.5 | No | Yes |
| 11 | 3.0 | 3.5 | Yes | No |
| 12 | 3.5 | 5.0 | No | Yes |
| 13 | 2.5 | 3.0 | No | Yes |
| 14 | 3.5 | 4.5 | Yes | No |
| 15 | 3.0 | 4.0 | Yes | Yes |
| 16 | 3.5 | 4.5 | No | Yes |
| 17 | 3.0 | 4.0 | No | Yes |
| 18 | 3.0 | 4.0 | No | Yes |
| 19 | 3.5 | 4.5 | No | Yes |
| 20 | 2.5 | 4.0 | Yes | Yes |
| 21 | 3.0 | 4.5 | No | Yes |
| 22 | 3.5 | 4.0 | No | Yes |
| 23 | 3.5 | 5.0 | No | Yes |
| 24 | 3.5 | 4.5 | No | Yes |
| 25 | 3.0 | 4.5 | Yes | Yes |
| 26 | 3.5 | 4.5 | No | Yes |
| 27 | 3.0 | 4.5 | No | Yes |
| 28 | 2.5 | 4.0 | Yes | Yes |
| 29 | 2.5 | 3.5 | No | Yes |
| 30 | 3.0 | 4.5 | No | Yes |
